# Dr. Barnardo and His Work

**Published:** 1903-01-31

**Authors:** 


### DR. BARNARDO AND HIS WORK.

WE do not know how many of our readers have paid attention to the National Waifs Association, better known as Dr. Barnardo's Homes, or how far they may have studied the movement by being present at one of the children's Christmas entertainments at the Royal Albert Hall. It would be a great gain to every philanthropic institution and to the whole of the workers who labour so zealously and successfully in the cause of philanthropy, if they would make it their business to become acquainted with the methods and work of the larger institutions amongst the general charities. In this way the best ideas of all the more active spirits in the field of charity might permeate the whole body, and so there should arise a resultant force which must make for progress all along the line. Dr. Barnardo is a general who has recruited an immense army of children from the waifs and strays of our national life. He has succeeded in attracting to his work thousands of philanthropic people whose contributions and support have helped him to save tens of thousands of young lives from misery, and to give them that start in life which has enabled them to take their place in the nation as useful citizens, both at home and in the colonies.

We often wonder how many people, even amongst the supporters of a great organisation like the National Waifs Association, realise the actual output of energy and intelligence on the part of the director, represented by the successful administration and continuous and wise extension of a work of this character. It would be well for London and Londoners, and well for Dr. Barnardo's homes, if everybody possessed of intelligence were to note these children's Christmas entertainments at the Royal Albert Hall, and to make a point of being present at at least one such entertainment. The Royal Albert Hall will hold 10,000 people, a fact which appals most organisers and warns them from its walls, when they contemplate bringing their work to the notice of the public. We have no doubt Dr. Barnardo, on the contrary, regards the Royal Albert Hall as all too small for his purpose. If so, we gain an insight into his character, which is instructive and helpful. Nothing is too large or responsible in the way of work for the young, in Dr. Barnardo's view, and yet no waif is too small or youthful to appeal to him and his co-workers. The tiniest mite when discovered will gain, without any hesitation or delay, the maximum of benefit which the National Waifs Association has in its power to bestow.

It requires other gifts, however, than those we have indicated to make an entertainment, even a children's Christmas entertainment, at the Royal Albert Hall an unqualified success. Dr. Barnardo must have something of the showman in his nature, and he might, had he chosen, we make no doubt, have been a successful manager of Drury Lane Theatre. At the entertainment at which we were present music formed a striking feature, but the orchestra and choir were kept well in hand, and the practical side of the work was brought prominently forward in a succession of "turns," as striking as they were happily conceived and executed. Dr. Barnardo has a hospital, for instance, Her Majesty's Hospital at Stepney, and he brought a whole ward into the programme, and exhibited some of its life and work, whilst a visit from Father Christmas, pleased the children and audience immensely. Then there was an exhibition of gun and cutlass drill by small boys from the Watts' Naval Training School, North Elmham, Norfolk, which would have done credit to any naval school, or for that matter to any ship's company in his Majesty's fleet. Again, in order to show the handicrafts taught the children in the schools and orphanages under Dr. Barnardo's control, a procession of young tradesmen bearing trophies of their skill and badges of their crafts paraded the hall. Sixteen trades were thus fully represented, including blacksmiths, box-makers, brush-makers, carpenters, engineers, harness-makers, mat-makers, printers, shoemakers, tailors, tinsmiths, wheelwrights, and aërated water makers, with bakers and cooks. The varied nature and extent of the numerous industries carried on at the Homes by the boy inmates could not have been more graphically or interestingly brought to the notice of the vast audience. It was a vast audience, by the way, for the Royal Albert Hall was crowded in every part—a triumph of organisation, seeing that most Londoners have more than enough to attract them elsewhere on a Saturday afternoon. The most popular and probably on the whole the most perfectly executed turn was that of some twenty little Jack Tars from the good ship *Jersey*, who sang a sailor song and executed certain hornpipes and dances with a precision and verve which were admirable. They deserved and, by request of H.R.H. the Princess Christian, who presided, they received an encore, to the great delight of everybody present. Nor was the girls' side of the work forgotten, for there was an illustrative exhibition of life and work in the girls' village homes, which represented groups of girls engaged in laundry work, cooking, dressmaking, art embroidery and needlework. This turn was carefully thought out and full of interest.

But Dr. Barnardo would not have met with the success he has done had he not always a strict eye to business on these occasions. A number of children of both sexes presented purses to the Princess in aid of the funds of the Young Helpers' League, medals and ribands of the distinguished order of Waif Service with silver badges of honour were conferred by the Princess upon officers and companions of the League, and the collection taken up to the sound of four cornets calling the audience to attention were all effectively managed. Nor was humour of a successful kind lacking, for the collection on the platform was started with gold in each plate. No one who has to do with the raising of money for charities could be present in the Royal Albert Hall, with eyes to see, and fail to be cheered, instructed and amused. That is why we again say that much benefit will accrue to individual workers, and to the cause of charity in the United Kingdom, if the active spirits of each institution make it their business to keep their eye upon the methods and work of all those responsible for other branches of benevolence than their own.

So much for Dr. Barnardo's Review Day. Now for his work in the aggregate: He has under his care, speaking in round numbers, 6,400 boys and girls, and 3,500 fresh cases were admitted to his homes in 1902, which maintained during that year 9,000 children. He has already rescued, trained and placed out in life nearly 50,000 orphan-waifs, and at least £200 per day is required to defray the cost alone of the food consumed by the children under his care. Dr Barnardo justly boasts that he never declines a destitute child, even if sick, afflicted and incurable, nor indeed any helpless infant. He admits freely to his homes, every week in every year from all over the Kingdom, something like 70 new cases, whilst without intermission his representatives and agents are searching out necessitous and neglected children in the slums of our great cities. We have often criticised Dr. Barnardo because he does not publish his accounts on the uniform system, or indeed upon any system which supplies information in sufficient detail to enable the cost and value of his work to be accurately appraised by those who know most about such matters. This is a weakness which may be due to the circumstance that the enormous burden of labour which must necessarily devolve upon Dr. Barnardo as the director of this enterprise, renders it impossible for him to appreciate, as he otherwise would do, the importance from a financial point of view of altering the system upon which his accounts are at present kept and published. To effect these changes in the accounts would be calculated however to bring an additional revenue of quite £10,000 a year to the Barnardo Homes. But this apart, we hold and feel that Dr. Barnardo deserves well of his day and generation, and that he is entitled to receive the generous confidence and monetary support of the thoughtful amongst his countrymen and countrywomen, whilst his claims upon the wealthy, subject to better accounting, are probably second to none in the world of charity.

